# Perceptual integration of head and eye cues to gaze direction in schizophrenia

**DOI:** 10.1098/rsos.180885

**Published:** 2018-12-12

**Authors:** Colin J. Palmer, Nathan Caruana, Colin W. G. Clifford, Kiley J. Seymour

**Affiliations:** 1 School of Psychology, UNSW Sydney, Sydney, New South Wales 2052, Australia; 2 Department of Cognitive Science, Macquarie University, Sydney, New South Wales 2109, Australia; 3 ARC Centre of Excellence for Cognition and its Disorders, Sydney, Australia; 4 School of Social Sciences and Psychology, Western Sydney University, Sydney, New South Wales 2150, Australia

**Keywords:** schizophrenia, visual integration, gaze perception, social cognition, theory of mind, Wollaston illusion

## Abstract

The perceptual mechanisms that underlie social experience in schizophrenia are increasingly becoming a target of empirical research. In the context of low-level vision, there is evidence for a reduction in the *integration* of sensory features in schizophrenia (e.g. increased thresholds for contour detection and motion coherence). In the context of higher-level vision, comparable differences in the integration of *sensory features of the face* could in theory impair the recognition of important social cues. Here we examine how the sense of *where other people are looking* relies upon the integration of eye-region cues and head-region cues. Adults with schizophrenia viewed face images designed to elicit the ‘Wollaston illusion’, a perceptual phenomenon in which the perceived gaze direction associated with a given pair of eyes is modulated by the surrounding sensory context. We performed computational modelling of these psychophysical data to quantify individual differences in the use of facial cues to gaze direction. We find that adults with schizophrenia exhibit a robust perceptual effect whereby their sense of other people's direction of gaze is strongly biased by sensory cues relating to head orientation in addition to eye region information. These results indicate that the visual integration of facial cues to gaze direction in schizophrenia is intact, helping to constrain theories of reduced integrative processing in higher-level and lower-level vision. In addition, robust gaze processing was evident in the tested participants despite reduced performance on a theory of mind task designed to assess higher-level social cognition.

## 1. Introduction

In the past decade, there has been increasing recognition that schizophrenia is associated with systematic differences in basic perceptual function, in addition to the hallmark positive symptoms (i.e. hallucinations and delusions), negative symptoms (e.g. apathy) and cognitive dysfunction. There is now a large body of research on perceptual function in schizophrenia, the majority of which has focused on visual processing. A key concept that has become useful in understanding the nature of visual processing in schizophrenia is that of *visual integration*, whereby elementary visual information is combined to form increasingly complex representations of the world [1]. For instance, a reduced ability to discern *contours* or *shapes* formed by adjacent visual elements, when these are presented among a cluttered background, has been replicated several times in groups with schizophrenia (e.g. [2–4]). Sensory integration deficits are also a potential explanation of differences in visual perception observed in schizophrenia across domains that include form, motion coherence and face perception [1,5–7].

A key challenge to understanding perception in schizophrenia is in identifying how abnormalities in sensory processing impact upon daily function. In this regard, a promising avenue of research is in how visual processing differences feed into higher-level *social-cognitive* and *social-behavioural* problems. Difficulties with maintaining interpersonal relationships, interacting with others in the community and the workplace, and social problem-solving can occur in schizophrenia, and may contribute to more general functional difficulties such as vocational success and the ability to live independently. The cognitive mechanisms that underlie social functioning, such as the perception of social cues (e.g. emotional face expressions) and reasoning about others' mental states (i.e. theory of mind), are thus becoming an increasing focus of research in this condition [8–10]. Interestingly, one study found a relationship between measures of *early visual processing* and *functional outcomes* that was strongly mediated by *social perception skills*, where the latter was measured in terms of the appropriate interpretation of others' verbal and non-verbal behaviours [11]. Thus, one hypothesis is that differences in how certain features of sensory input are processed in the visual system in schizophrenia, in particular those that carry information about other people, contribute to impairments in social behaviour.

A key example of how visual integration deficits might impact upon social function is the processing of *facial* cues important to communication and interaction. Visual integration can be considered at both the level of *specific* facial features (e.g. binding the visual elements that form the shape of the mouth) or *configurations* of facial features (e.g. identifying a facial expression that is conveyed by both the eyes and the mouth together). Several studies investigating *face recognition* in schizophrenia have found evidence for reduced integrative processing of face information, reflected in how recognition performance varies from controls as integrative processing is disrupted by image manipulations like fragmentation and spatial inversion [12–14]. For example, in one task, participants were shown pairs of unfamiliar face photographs and asked to indicate whether it was the same person depicted in each photograph or not [14]. Recognition was compared between conditions in which the photographs were presented upright compared to when they were presented upside down, based on the idea that the latter may impair performance by disrupting the typical spatial relationship between face features. Participants with schizophrenia performed worse on this task than controls, but the disruptive effect of inverting the face was less pronounced compared to the control group, one interpretation of which is a lesser reliance on (or sensitivity to) the configuration of face features when performing face recognition. The evidence is currently mixed, however, as other studies have found that manipulations intended to disrupt integrative processing produced similar effects on face recognition in schizophrenia to those observed in controls, despite the general deficit in face recognition observed in this condition (see [15], for review). The perception of *emotional facial expressions* is another aspect of face processing that is impaired in schizophrenia, and associated with a spatial distribution of neural responses that differs from controls [8,16]. Studies of emotional face perception that use spatial inversion of the face image to disrupt integrative processing again provide mixed evidence that this may contribute to worse performance in correctly labelling emotional facial expressions [17–19]. Research that complements these findings with other methods for assessing visual integration in face perception may help to bring clarity to this area.

**Figure 1.**
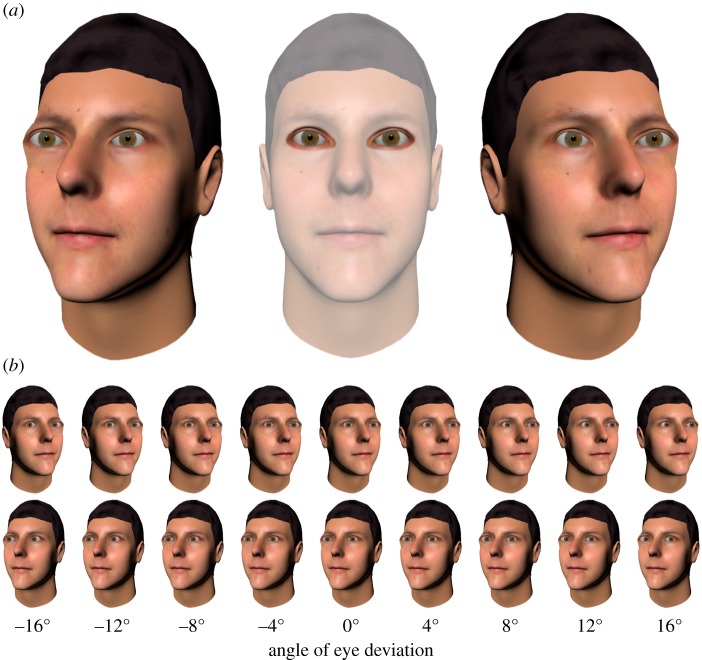
(*a*) Wollaston face stimuli, in which *identical eye regions* appear to be looking in different directions depending on the orientation of the head. In each of these images, the same eye region (highlighted in the central face) is presented within different head regions using image editing. (*b*) Wollaston face stimuli were generated with a range of eye deviations for head orientations 20° left and 20° right. These images are for one of six facial identities used in the experiment.

Another aspect of face perception that relies upon visual integration is our sense of *where other people are looking*. The role of visual integration in gaze perception is vividly demonstrated by the Wollaston illusion [20], in which *identical eye regions* appear to be looking in different directions depending on the orientation of the surrounding head (figure 1*a*). Thus, our sense of where other people are looking relies fundamentally on the integration of different facial cues, relating to sensory features of both the eye region and the head region [21,22]. The sensory features extracted from the eye region likely relate to how the reflectance characteristics of the iris and sclera determine the luminance distribution across this region, while the features conveyed by the head region likely include nose deviation and head contour (see [22], for review). Recent research in visual psychophysics has exploited the Wollaston illusion to systematically investigate the integration of head and eye cues to gaze direction in the visual system [23–26]. In this context, the head region acts as a direct cue to gaze direction, drawing the perceived gaze direction away from the veridical direction of the eyes and towards the orientation of the head.

There is currently only limited data on the perception of other people's direction of gaze in schizophrenia, with some studies reporting differences in gaze perception relative to the general population (e.g. [27,28]) and others not (e.g. [29]) (reviewed in §4.2). Interestingly, Tso and colleagues [30] report that differences in gaze perception in a clinical sample correlate with performance on a low-level measure of visual integration (contour perception). It is yet to be directly examined, however, whether perceived gaze direction in schizophrenia is characterized by reduced integration of sensory cues. This is an important question to address, as the perception of gaze direction is crucial to daily social functioning. For instance, our sense of others' direction of gaze underpins in part how we infer the intentions and mental states of other people (i.e. theory of mind; [31]), and coordinate our own actions or focus of attention with theirs [32]. In this way, sensitivity to others' eye gaze is important to cooperative dyadic or group behaviour, reliable communication, and developmental processes such as word–object association learning [33,34]. An apparent lack of sensitivity to others' eye gaze, from early in development, is a core diagnostic feature of autism spectrum disorder, a condition that is associated with social-cognitive impairments that overlap with those now implicated in schizophrenia (e.g. theory of mind; [35]). The perception of other people's gaze direction therefore presents as an aspect of visual processing for which a disturbance in the integration of sensory features has the potential to cascade into more general problems with social function.

Here we conduct a psychophysical study of the perception of gaze direction in adults with schizophrenia, in a step towards probing the fundamental perceptual mechanisms that underlie social experience in this condition. Specifically, we examined the perception of Wollaston faces, in which the eyes and head each provide independent cues to gaze direction. We compare across faces in which the eye direction is held constant, while the head region (i.e. the surrounding sensory context) is manipulated. We perform detailed computational modelling of the participant data to quantify individual differences in the visual integration of facial cues to gaze direction. This approach differs from past studies of integrative processing of facial information in schizophrenia by quantifying the influence of distinct facial cues on a perceptual outcome (here, the perceived gaze direction of the face), rather than aiming to disrupt integrative processing via image manipulations like spatial inversion. We find that adults with schizophrenia exhibit a perceptual effect whereby their sense of other people's gaze direction is strongly biased by sensory cues relating to head orientation in addition to eye region information. Intact gaze processing is evident in these participants despite impaired performance on a theory of mind task, which was included to confirm that the recruited sample of schizophrenia patients presented with the social-cognitive difficulties reported previously for this population [8]. These results indicate that visual integration occurs robustly in schizophrenia in the domain of eye gaze perception, and thus this mechanism is unlikely to contribute significantly to deficits in social cognition. Moreover, these data on cue integration in the context of gaze perception may be important for constraining theories of integration deficits in schizophrenia in lower-level and higher-level vision.

## 2. Material and methods

### 2.1. Participants

Participants were 22 adults with a diagnosis of schizophrenia (*M*_age_ = 51.9, s.d. = 8.7, 13 males) and 27 healthy controls (*M*_age_ = 41.3, s.d. = 14.6, 14 males). The recruited groups were closely matched in gender (Chi square (1, *n* = 49) = 0.048, *p* = 0.83), though the group with schizophrenia were somewhat older on average than the control group (*t*_43_ = 3.1, *p* < 0.01). Most participants completed the National Adult Reading Test (NART) as a measure of premorbid intelligence, and the full scale scores of this measure were closely matched between the participants with schizophrenia (*M* = 107, s.d. = 10, *n* = 21) and controls (*M* = 110, s.d. = 7.5, *n* = 20), *t*_37_ = 1.2, *p* = 0.22. Two further participants with schizophrenia completed the experiment but were excluded prior to data analysis as they failed to complete the task correctly.

Patients were recruited from the Volunteer Schizophrenia Research Register of the Australian Schizophrenia Research Bank [36] and the Macquarie Belief Formation Volunteer Register. Diagnosis of Schizophrenia or Schizoaffective Disorder was confirmed using the Diagnostic Interview for Psychosis [37]. This was completed by either a Clinical Neuropsychologist with over 10 years of experience working with patients or a Clinical Psychologist, both with postgraduate qualifications in conducting clinical diagnoses for schizophrenia. Clinical demographics were recorded, and symptom severity was assessed using the Scales for Assessment of Positive and Negative Symptoms (SAPS & SANS; [38,39]). Clinical ratings were completed by a PhD-level research assistant who was trained by the Clinical Neuropsychologist and had initial clinical ratings double-scored during training. All patients were on stable doses of antipsychotic medication and each participant reported normal or corrected-to-normal vision. The mean age of diagnosis was 26.1 years (s.d. = 9.8).

Healthy controls were screened using a structured interview to assess for potential affective, psychotic or substance abuse disorders. This was based on the screening modules from the Structural Clinical Interview for Axis 1 Disorders previously outlined under DSM-IV (SCID-1) [40]. Exclusion criteria for both groups included current or past (within past five years) central nervous system disease or head injury, and having less than eight years of formal education. All participants gave their written informed consent, which was approved by Macquarie University's Ethics Committee (Ref# Caruana5201200021).

### 2.2. Experimental task

Participants completed a task designed to quantify the relative influence of the head and eye direction in a face image on perceived gaze direction. In this task, participants viewed Wollaston-illusion face images (like that depicted in figure 1*a*) and judged the direction in which these faces were looking. Face images with a range of different eye directions were presented in the context of either leftwards- or rightwards-oriented head regions (figure 1*b*). The purpose of using Wollaston-illusion face images was so that identical eye regions could be presented in the context of different head regions. This allowed us to quantify, for each participant, how strongly head information drew the perceived gaze direction away from the veridical direction that the eyes were pointing (detailed further in §2.3).

The Wollaston-illusion face images were generated using 3D graphical modelling and image editing software, following a procedure similar to that employed in previous studies (e.g. [25]). Specifically, 3D face models and skin textures were generated using Face-Gen Modeller 3.5, and imported into the scene-based modelling program Blender 2.70. Using a custom Blender script, we generated face images with precise control over the rotation of the eyes within the head, and the rotation of the head relative to the viewer. Images of a forward-facing head were generated with eye direction varying between 16° left and 16° right in 4° intervals. Images were also generated with head direction 20° left or 20° right. To create images that would induce the Wollaston illusion, the *eye regions from the forward-facing head* were cropped and merged with the *head regions from leftwards- and rightwards-rotated faces* using Adobe Photoshop. This resulted in the set of images displayed in figure 1*b*. Importantly, this meant that the eye regions in the faces were identical between the ‘left head’ and ‘right head’ conditions, such that these conditions differed only in the head-region information available. Thus, any difference in perceived gaze direction between these conditions would be a consequence of head region information.

For each combination of head direction and eye direction, participants completed 12 trials consisting of 6 different facial identities each repeated twice. This amounted to 216 trials in total, presented in a random series. Each image was presented for 1500 ms, after which the task paused on a blank screen until the participant made a response. Trials were also separated by a further 500 ms blank period. Participants were asked to classify each face as looking to their left, to their right, or directly towards them, by pressing the appropriate key on a keyboard.

Participants completed the task on a 26-inch monitor (1920 × 1080 pixel resolution), with a viewing distance of 80 cm. The face stimuli were presented on screen at approximately life-size, as defined by an inter-pupillary distance of 6.3 cm [41].

### 2.3. Psychophysical modelling

In order to quantify the effect of head information on perceived gaze direction for each participant, we fit a model of categorical responding to the individual participant data (described previously in [42]). This model estimates the probability of a given face stimulus being perceived as looking direct, by accounting for (i) the position of category boundaries used when making this judgement (i.e. reflecting the range of eye deviations that the participant categorizes as looking either ‘leftwards’, ‘direct’, or ‘rightwards’), and (ii) variability in participants' categorization of stimulus gaze direction, which might relate to either noisy sensory representations or other sources of response variability. The model of perceived gaze direction is formulated as follows:

$$p(\mathrm{Direct})=\int_{P_{0}-(w/2)}^{P_{0}+(w/2)}G(\theta_{\mathrm{stim}},\;\;\sigma_{\mathrm{rep}})\;d\theta ,$$

where *p*(Direct) is the probability of the participant categorizing a stimulus with eye deviation *θ*_stim_ as looking direct. This probability is calculated by integrating the normal distribution, *G*, between the category boundaries for direct gaze. The normal distribution, *G*, is centred on the stimulus eye deviation, and has standard deviation *σ*_rep_, capturing variability in participant categorization. The category boundaries are described in terms of the midpoint of the category, *P*_0_, and the width of the category, *w*. See [42] for a full description of this model.

**Figure 2.**
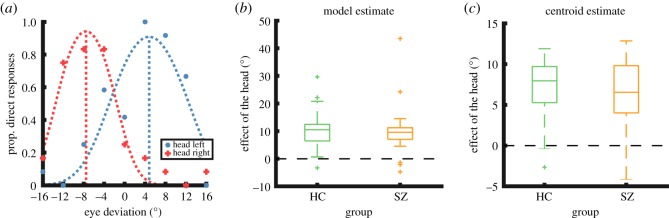
(*a*) An example of individual participant data, showing the proportion of direct responses as a function of stimulus eye deviation. Negative values on the *x*-axis indicate leftwards eye deviations. Face stimuli with head orientation 20° left and 20° right are plotted separately. The dotted lines show a psychophysical model of perceived gaze direction fitted to the participant data for each condition. The average difference between the eye deviation at which the peaks of the two model functions occur (i.e. the *cone model half-difference*) is a measure of the effect of head orientation on perceived gaze direction. This specific participant shows a cone model half-difference of approximately 6°, indicating that head orientations of 20° tend to draw the perceived gaze direction about 6° away from veridical. (*b*) The estimated effect of head orientation on perceived gaze direction, for healthy controls (HC) and participants with schizophrenia (SZ). Here the effect of head orientation is quantified in terms of the cone model half-difference obtained by fitting the model of perceived gaze direction to the data. Both groups exhibit a strong effect of head orientation on perceived gaze direction. See §3.1 for statistics. (*c*) The estimated effect of head orientation on perceived gaze direction, quantified by finding the average difference between the centroids of the data for the two head orientation conditions (i.e. the *centroid half-difference*). These data support the same conclusions as the modelling approach. See §3.2 for statistics.

The model was fit to the *proportion of direct responses* across eye deviations, resulting in a cone-shaped function like those shown in figure 2*a*. The model was fit to the data separately for each head condition (i.e. leftwards head and rightwards head), and the eye deviation at which the maximum of the function occurred was taken as an estimate of perceived ‘direct’ gaze. The model was fit to the data by minimizing the sum of squared errors using the *fminsearch* function in MATLAB, allowing three parameters to vary: *P*_0_, *w*, and *σ*_rep_.

Intuitively, given that the eye deviations are identical across the two head conditions, if the head information has no effect on perceived gaze direction, then the estimate of perceived ‘direct’ gaze (i.e. the maximum of the model fit) would also be identical between conditions. A *cone model half-difference* value was thus calculated as a measure of the average effect of head information on perceived gaze direction. This measure was taken as the difference between the estimate of perceived ‘direct’ gaze for the two head conditions, divided by 2, thus representing the average extent to which head orientation pulled perceived gaze direction away from that indicated by eye region information alone. The cone model half-difference (computed for each participant) was compared between the participant groups to test for a difference in the integration of head orientation and eye deviation in perceived gaze direction.

### 2.4. Centroid method

The model of gaze perception described in the previous section generally fit the data very well (see §3.1). However, when participants were either noisy in their responding, or tended only to categorize the direction of gaze as ‘direct’ for more extreme eye deviations, the data did relatively little to constrain the model. This occurred only for a handful of participants, but tended to result in large cone model half-difference values, which were likely overestimates of the effect of the head on perceived gaze direction.

While the model is a principled method for estimating the strength of the Wollaston illusion, we also employed an alternative *centroid* method to ensure that the results were robust across different methods for quantifying the illusion. For each participant, we calculated the centroid of their data across eye deviations, separately for each head condition. The centroid was calculated by summing the proportion of direct responses for each eye deviation multiplied by the corresponding eye deviation value, divided by the sum proportion of direct responses across eye deviations. The average difference between the centroids for each head condition, i.e. the *centroid half-difference*, was taken as an estimate of the effect of the head on perceived gaze direction.

Given that the centroid represents the midpoint of the data, these values are constrained within the range of eye deviations tested (i.e. between 16° left and 16° right in the present study). Thus, the centroid half-difference is more liable to *underestimate* the effect of the head on perceived gaze direction compared to the model described in §2.3. In this way, the centroid method complements the modelling approach by providing a more conservative estimate of the strength of the Wollaston illusion.

### 2.5. Theory of mind task

A core aspect of social cognition that has been a focus of schizophrenia research is the ability to infer or reason about other people's mental states in a typical way, usually referred to as theory of mind or mentalising [8]. A measure of theory of mind was included in the present study to verify that the recruited sample of schizophrenia patients presented with impaired social cognition. We used the task reported in [43], which itself is adapted from [44].

The task assessed reasoning about other people's mental states by presenting participants with written stories and asking them to report on the beliefs of the characters in each story. This included both ‘first order’ theory of mind (e.g. understanding that a character can have a false belief) and ‘second order’ theory of mind (e.g. understanding one character's beliefs about another character's beliefs). Participants were also asked questions about basic facts contained in each story to assess general comprehension. There were two first-order and two second-order stories (i.e. four stories in total). To assess theory of mind, participants were asked questions both about how a character was likely to act next in the story (scored as 1 or 0 for correct or incorrect responses) and to explain why they would act in this way (scored as 2, 1, or 0 for correct, partially correct, or incorrect responses). The comprehension questions were scored as 1 or 0 for correct or incorrect responses. These scores were summed across both stories within each story condition (i.e. first-order, second-order), resulting in a maximum score of 6 for theory of mind questions and 2 for comprehension questions in each story condition.

## 3. Results

### 3.1. Psychophysical modelling

The model fit the participant data very well, with the mean variance explained by the model 91% for controls (s.d. = 15%) and 92% for participants with schizophrenia (s.d. = 9%). The mean variance explained by the model did not differ significantly between groups, *t*_47_ = 0.28, *p* = 0.78.

Both groups showed a strong effect of head orientation on perceived gaze direction (figure 2*b*). This was indicated by a mean cone model half-difference that was significantly greater than zero for both participants with schizophrenia, *t*_21_ = 4.87, *p* < 0.001, Hedges *g_rm_* = 1.44, and controls, *t*_26_ = 8.15, *p* < 0.001, Hedges *g_rm_* = 2.19 (one-sample *t*-tests). The mean cone model half-difference for participants with schizophrenia was 9.94° (s.d. = 9.58°) and for controls 10.68° (s.d. = 6.81°), indicating that the 20° head orientations used in this experiment tended to shift the perceived gaze direction by about 10° on average. This is a large effect, as differences in gaze direction of 10° are in general very easy to discriminate.

There was no significant difference between the groups in the strength of head orientation on perceived gaze direction, *t*_47_ = 0.31, *p* = 0.76 (independent-samples *t*-test). A Bayesian independent samples *t*-test was also performed to quantify the evidence for the null hypothesis relative to the alternative hypothesis. A Cauchy prior width of 0.7 was used. This test indicated that there was ‘moderate’ evidence in the data that the groups showed the same effect of head orientation on perceived gaze direction, BF_01_ = 3.36. This Bayesian analysis was performed using the JASP software package [45], and interpreted with respect to guidelines suggested in [46] and [47].

### 3.2. Centroid method

The centroid method for estimating the strength of the Wollaston illusion showed the same pattern of results as the psychophysical modelling, with both participant groups showing a strong effect of head orientation on perceived gaze direction (figure 2*c*).

The mean centroid half-difference was significantly greater than zero for participants with schizophrenia, *t*_21_ = 6.62, *p* < 0.001, Hedges *g_rm_* = 1.96, as well as controls, *t*_26_ = 10.30, *p* < 0.001, Hedges *g_rm_* = 2.76 (one-sample *t*-tests). The mean centroid half-difference for participants with schizophrenia was 6.13° (s.d. = 4.34) and 6.96° (s.d. = 3.51) for controls. There was no significant difference between the participant groups, *t*_47_ = 0.74, *p* = 0.46, BF_01_ = 2.79. This Bayes factor value indicates ‘anecdotal’ evidence for the null hypothesis.

### 3.3. Theory of mind task

Participants with schizophrenia did reveal a specific difficulty with theory of mind compared to controls for both first- and second-order stories. This was demonstrated by a significant group condition interaction, *F*_1,47_ *=* 8.76, *p* < 0.001, in which the schizophrenia group performed significantly poorer than controls for both first order theory of mind questions, *t*_28.06_ = −2.85, *p* < 0.001, and second order theory of mind questions, *t*_47_ = −4.17, *p* < 0.001, but not on comprehension questions based on the same stories (*p*s > 0.16). Descriptive statistics for this task are presented in table 1.

**Table 1.** Means and standard deviations for theory of mind task.

stories	schizophrenia group	control group
theory of mind, first order	4.27 (1.96)	5.56 (0.89)
theory of mind, second order	3.00 (1.85)	4.93 (1.38)
comprehension, first order	1.73 (0.55)	1.89 (0.32)
comprehension, second order	1.91 (0.29)	2.00 (0.00)

## 4. Discussion

Our sense of where other people are looking relies fundamentally on the visual integration of different facial cues to gaze direction [20–22,24–26]. Here we find that adults with schizophrenia exhibit a strong Wollaston illusion, in which the perceived gaze direction is dependent not only on where the eyes are pointed, but also on the orientation of the head. This indicates that the integration of different sensory cues to gaze direction occurs robustly in schizophrenia.

### 4.1. Visual integration in schizophrenia

The notion of reduced integrative processing in schizophrenia is appealing in its potential to explain differences in performance across perceptual domains, and the possibility that the function of visual mechanisms are indicative of neural processing differences relevant for understanding higher-level functions as well [5]. There is evidence for disrupted integrative processing in schizophrenia for low-level aspects of vision, reflected, for example, in reduced sensitivity to the presence of contours in noise or dot-motion coherence [1,5–7]. The extent to which such findings reflect disrupted *integration* rather than other visual processing differences, and a *generalized* rather than *specific* processing difference, is still an active area of investigation. For example, a more recent study found that individuals with schizophrenia differed in judgements about the *average orientation* of a set of gratings (i.e. reflecting a form of global or integrative processing), but did not differ from controls in similar tasks that tested sensitivity to the average direction of motion or object size [48]. This highlights the value of investigating visual integration across perceptual domains. Similarly, findings of robust integration are important in this area for defining the extent or distribution of impaired versus intact integrative processing across the brain (e.g. in low-level versus higher-level vision). Relevant to the focus of the present study, the visual representation of other people's direction of gaze occurs in higher-level visual pathways in the temporal cortex. Specifically, single-unit recording studies in macaque monkeys and functional neuroimaging in humans indicate the existence of cell populations in anterior superior temporal sulcus with responses that are selective to the gaze direction of a seen face (e.g. leftwards versus rightwards gaze direction), and in some cases appear to incorporate both head and eye direction information [49–53].

There is evidence for differences in face perception in schizophrenia that can be related to the integration of facial features, including the recognition of personal identity and emotional expressions [8,15,16]. Some studies have employed image manipulations such as spatial inversion or fragmentation to disrupt integrative processing of the face. For instance, inversion of the face alters the typical spatial configuration of individual face features and the spatial relationship between them. Thus, the idea is that performance will be disrupted less by this manipulation in schizophrenia if there is a tendency towards reduced integrative processing of the face. These studies have reported mixed evidence that such manipulations impact upon identity recognition (see [15], for review) and the perception of emotional facial expressions [17–19]. Butler and colleagues [54] also find that when matching pairs of faces, individuals with schizophrenia show similar changes in performance to controls when the face images are either inverted or disrupted by other featural and configurational manipulations (e.g. altering the spacing between face features), suggesting intact integrative processing of faces. The methodological approach of the present study differs from past research in assessing perceptual integration by quantifying the relative influence of distinct facial cues to gaze perception (i.e. head-region cues and eye-region cues), rather than examining how performance changes as integrative processing is impeded. In a sense, the present approach pits context-sensitive and feature-based processing against one another, where the head orientation in the Wollaston illusion acts as a sensory context that draws perceived gaze direction away from that which is indicated by the eyes. Thus, integrative processing of face features is expected to result in a shift in perceived gaze direction away from the veridical direction of the eyes (as observed in both participant groups in the present study), while reduced integrative processing is expected to result in perceived gaze direction being relatively unaffected by head direction.

The present results complement a previous study related to integrative processing of eye gaze in schizophrenia. Tso and colleagues [30] report that performance on a contour detection task in a sample of people with schizophrenia correlated with their judgements regarding another person's direction of gaze; in particular, worse performance on the detection task, which required visual integration of local features to locate the target contour among distractor elements, was associated with a less sharp transition between classifying angles of eye deviation as direct or averted as the angle of eye deviation increases (i.e. there was more uncertainty in making this categorization). However, there was no significant correlation between measures of gaze perception and two other measures of low-level visual integration, namely a different contour detection task (where difficulty is added to the task by increasing the density of the elements rather than increasing jitter in the orientation of the elements) and dot-motion coherence. The approach of the present study differs notably from that of Tso and colleagues [30] in that we examine the integration of eye and head cues by quantifying their relative contribution to perceived gaze direction (i.e. investigating integration at the level of facial cues), while this previous work examines the association between distinct measures of lower-level visual processing (i.e. contour perception) and gaze perception.

A common approach to examining visual integration is with the use of *visual illusions*, which often arise when a part of the sensory input is misperceived as a result of the broader sensory context (e.g. [55]). A range of visual illusions have been examined in schizophrenia. A recent review concluded that most studies had found reduced susceptibility to visual illusions in schizophrenia, consistent with the notion of disrupted integrative processing of visual elements [56]. These results differed between different cases, however; for instance, there is evidence for reduced susceptibility to the hollow-mask illusion in schizophrenia, but not to brightness illusions. This highlights the value of examining susceptibility to perceptual illusions that reflect processing in different brain pathways (e.g. higher versus lower-level sensory processing). The present study adds to this literature by demonstrating an intact Wollaston illusion in adults with schizophrenia.

### 4.2. Perception of gaze direction in schizophrenia

Does the perception of gaze direction occur differently in people with schizophrenia? Some research indicates a tendency towards a wider ‘cone of direct gaze’ in this condition, whereby a wider range of eye deviations around direct are reported as looking at oneself [27,28]. A recent study, however, found similar discrimination of gaze direction in schizophrenia and controls, across a range of gaze deviations, when using a response method designed to be less *self-referential* [29]. Specifically, rather than participants being asked to report whether a face was looking at them or not, they reported whether the face looked straight ahead, leftwards, or rightwards. Hooker & Park [27] also examined judgements about gaze direction for briefly-presented faces in which the inner facial features, including the eyes, were absent (i.e. only the face outline and hair are presented). Here, participants with schizophrenia were also more likely than controls to report that the faces were looking at them, despite the lack of eye region information. Together, these results could be accounted for if the discrimination of eye direction is *typical* in schizophrenia, but what differs is the (more cognitive) categorization of whether one is the focus of another person's attention or not, whether this is indicated by eye direction or by the mere presence of a face.

Other initial evidence for intact early processing of gaze direction comes from a study of unconscious processing of gaze stimuli [57]. In this paradigm, *continuous flash suppression* is used to suppress visual awareness of a face stimulus that is presented to one eye. For both people with schizophrenia and healthy controls, faces with *direct gaze* break out of suppression earlier than faces with *averted gaze*, suggesting a similar processing and prioritization of gaze direction prior to visual awareness. Research has also examined how the *spatial focus of attention* is reflexively guided by other people's direction of gaze; while some studies have found reduced cuing of attention in schizophrenia compared to controls on the basis of pictorial eye cues [58,59], research using photographic face images has indicated that this mechanism is largely intact in schizophrenia [29,60–62].

In another recent experiment, we examined *sensory adaptation* to gaze direction in the same group of individuals with schizophrenia reported in the current paper [63]. The psychophysical effects of repeated exposure to faces with averted gaze can be modelled in terms of gain control mechanisms operating on gaze-selective sensory channels in the visual system. Specifically, changes in perception following adaptation may be indicative of both channel adaptability and divisive normalization processes in the sensory coding of gaze direction [64,65]. Individuals with schizophrenia exhibited robust perceptual after-effects following adaptation to averted gaze, and a modelling analysis of these psychophysical data indicated evidence for both channel adaptation and divisive normalization processes operating robustly in the context of gaze perception.

In sum, and including the present findings, the emerging, but still limited, literature on gaze perception in schizophrenia suggests that many early mechanisms are intact in this condition. This includes the discrimination of different horizontal eye directions, the integration of head and eye cues to gaze direction, gain control mechanisms reflected in the effects of recent stimulus history on perceived gaze direction, the apparent prioritization of direct over averted eye gaze prior to conscious awareness, and the spatial re-direction of attention on the basis of where other people are looking.

Moreover, in the present study, robust processing of gaze cues occurred in the schizophrenia group despite these individuals exhibiting social-cognitive difficulties, as reflected in their performance on a theory of mind task. Impaired performance on higher-order social cognitive tasks, including the theory of mind measure employed in the present study, has been reported previously in the literature [8,43,44]. The theory of mind measure was included in the current study to verify that the recruited sample displayed the expected social-cognitive difficulties. While eye gaze processing can play an important role in higher-order social cognition (e.g. when inferring other people's beliefs and intentions based on their behaviour; [31]), the present findings of a robust Wollaston illusion in schizophrenia suggest that integration of face cues to gaze direction is a mechanism that is unlikely to contribute significantly to social-cognitive difficulties in this condition.

### 4.3. Conclusion

This is the first study to assess a fundamental perceptual mechanism in the perception of eye gaze direction in schizophrenia, namely the integration of head and eye cues. This adds to a nascent body of literature on the perception of gaze direction in schizophrenia, which forms an important complement to research on more cognitive aspects of gaze processing, such as mentalising and attentional cueing [8,31,32]. Indeed, in the present study, intact gaze processing in the schizophrenia group was apparent despite reduced performance relative to controls in a theory of mind task. In addition, our finding that the perceptual integration of head and eye cues to gaze direction occurs robustly in schizophrenia helps to constrain theories of reduced visual integration in this condition. Specifically, these results suggest that integration at the level of facial cues to gaze direction, which is likely mediated by higher-level visual pathways in the temporal lobe (e.g. anterior STS; [51]), occurs typically in schizophrenia, while past findings suggest that lower-level visual perception [1,5–7] and potentially aspects of face perception such as identity recognition [15] may be associated with reduced integrative processing of visual features.

## Data accessibility

Data are accessible at https://osf.io/wyrns/?view_only=fda6aa3bd0f5401db8a67ca9ee8876ce.
